# Alcohol Use Disorder (AUD) in New Jersey (NJ): Disparity in Treatment

**DOI:** 10.7759/cureus.43286

**Published:** 2023-08-10

**Authors:** Bolaji Yoade, Oluwafemi Akinbode, Olubusola Olatunji, Olufemi Popoola, Oluwatoyin Busari, Nkolika Odenigbo, Irina Kogan, Stanley Nkemjika

**Affiliations:** 1 Public Health, Regis College, Weston, USA; 2 Family Medicine, American Family Care, West Orange, USA; 3 College of Health Sciences & Human Services, North Kentucky University, Kentucky, USA; 4 Neiswanger Institute of Bioethics and Healthcare Leadership, Loyola University, Illinois, USA; 5 Psychiatry, University Hospitals Cleveland Medical Center/Case Western Reserve University, Cleveland, USA; 6 Psychiatry and Behavioral Sciences, Interfaith Medical Center, Brooklyn, USA

**Keywords:** treatment, nj, sociodemographic, disparity, alcohol use disorder

## Abstract

Alcohol use disorder (AUD) continues to be a threat to public health due to the associated morbidity, mortality, and social and economic impacts. AUD accounts for greater than 85,000 deaths annually in the United States and greater than 1500 deaths annually in New Jersey (NJ). Despite these associated burdens, the treatment of AUD remains unequal among the population, and it is important to identify the factors influencing the disparity in defined population groups such as NJ to drive the appropriate intervention. Data were retrieved from the 2018 Treatment Episode Data Set-Discharges (TEDS-D) of the United States Substance Abuse and Mental Health Services Administration (SAMHSA). Logistic regression analysis was used to predict the odds of receiving treatment based on socioeconomic factors and the type of treatment received. Compared to Asian or Pacific Islanders in NJ, the American Indian [odds ratio, OR=2.12, 95% confidence interval, CI: 1.95-2.31] has the greatest odds of receiving treatment for AUD, followed by the Black or African American [OR=1.70, 95% CI: 1.65-1.75], the Alaska Native [OR=1.67, 95% CI: 1.42-1.96], and then the White [OR=1.31, 95% CI: 1.12-1.52]. Those who are retired or on disability [OR=0.88, 95% CI: 0.82-0.94] have lower odds of receiving treatment than those on salary or wages. Those with AUD in NJ have a lower odd of receiving detoxification treatment in a 24-h hospital inpatient setting [OR=0.88, 95% CI 0.82-0.95] and a higher odd of receiving detoxification treatment in a 24-h service, free-standing residential setting when compared to the treatment received in a rehabilitation/hospital (other than detoxification) setting.

This study shows that disparity exists in relation to the type of treatment received and the setting of treatment for AUD in NJ in addition to disparity based on the sociodemographic factors.

## Introduction

Alcohol is a psychoactive substance whose harmful use continues to be of significant public health importance due to the resultant burden of disease with striking social and economic consequences [1]. The harmful use of alcohol is responsible for most preventable deaths worldwide and has also been attributed to the cause of a range of behavioral and mental disorders [1-2]. In the United States, with a minimum legal drinking age of 21 years, about 87,798 alcohol-attributable deaths occur annually, with 2.5 million years of potential life lost, resulting in an estimated economic burden of $249 billion annually [2-3]. In New Jersey (NJ), the annual alcohol-attributable deaths are 1,754, and the annual number of years of potential life loss of 50,856 [2]. However, there remains a dearth in literature regarding the treatment infrastructure of the state and the attributes of residents seeking access to care.

Given the social and economic burden of AUD, it is crucial that people suffering from AUD are adequately treated and supported, and there is a body of evidence to substantiate the beneficial effect of receiving treatment for AUD. In a study conducted by Bold et al. (2017) on alcohol-dependent women in NJ, treatment of AUD was linked to an improvement in the physical, psychological, and social domains of life, including an overall improvement in the quality of life [4]. Nonetheless, existing literature has examined the treatment disparity in AUD, and the reports on their findings remain inconsistent. In a longitudinal study conducted by Mulia et al. (2014), it was reported that ethnic minorities have lower odds of getting an alcohol treatment compared to Whites over the five-year study period, with the most striking disparity found between the Whites and Hispanics [5].

Furthermore, Native Americans are two times more likely to get treatment for AUD, although it was reported as not statistically significant [5], thus requiring further investigation. Conversely, it was reported by Saloner and Lê Cook (2013) that Native Americans are less likely to complete treatment for AUD when compared to Whites [6]. Like Mulia et al. (2014), Saloner and Lê Cook (2013) in their study reported that Blacks and Hispanics are less likely to finish the treatment for AUD compared to Whites regardless of the setting where the treatment is being implemented [6]. This is substantiated by Guerrero et al. (2013), who also reported that Blacks and Latinos are less likely to complete AUD treatment [7], and these disparities in treatment completion could be attributed to various factors, including socioeconomic class, level of education, and employment status [6]. Palzes et al. (2022) examined whether the increased utilization of telehealth during the pandemic would cause a change in the disparities in AUD treatment [8]. Although increased initiation of treatment was recorded during the post-pandemic era compared to the pre-pandemic era, the racial and ethnic disparities in the treatment of AUD persist [8].

According to a study conducted by Nkemjika et al. (2022) on the disparity in substance use treatment in the tri-state area (NY-NJ-CT), it was reported that treatment rate completion is higher in New York when compared to NJ [9]. Due to the various concerns surrounding the disparities in the treatment of AUD in the United States, the inconsistencies in the reported results, and the literature evidence that suggests a disparity in treatment completion in NJ when compared to New York, this study seeks to explore the disparity that exists for AUD treatment in NJ. The aim is to identify the social and demographic factors contributing to the treatment disparity of AUD in NJ and suggest possible interventions to help alleviate any observed disparity.

## Materials and methods

The study sample (n=969788) was collected from the Treatment Episode Data Set-Discharges (TEDS-D) which is a standardized data system that serves as storage for the substance abuse treatment data collected by the states [10]. Data were retrieved for clients discharged from substance use treatment in 2018 [10]. The data collected include demographics (including age, sex, marital status, and race), substance abuse use, employment status, the primary source of income or support, the type of treatment received, and the setting of treatment for AUD. The sample was stratified based on clients who were managed for AUD or not.

The primary endpoint or the dependent variable is the “receipt of treatment for AUD” while the independent variables are the patient’s race (Alaska Native, American Indian, Asian or Pacific Islander, Black or African American, White), age group (12-24, 25-49, >50 years), the marital status (never married, now married, unemployed), employment status (full-time, part-time, unemployed, not in labor force), education (number of years in school/level of education) (less than one school grade, Grades 9-11, Grade 12 or GED, 1-3 years of college, 4 years of college), the primary source of income (wages or salary, public assistance, retirement or pension or disability, other), type of treatment received, and the setting of the treatment (detox 24 h hospital inpatient, detox 24 h free-standing residential, rehab, or residential hospital-non detox).

Using the SAS 9.4 statistical software (SAS, Cary, NC), we conducted a logistic regression to determine the odds of getting treatment for AUD based on the independent variables. Age 12-24 years, never-married status, unemployed status, grade 8 or less education status, salary or wages, Asian or Pacific Islander, and treatment received in a rehabilitation/residential hospital (other than detoxification) setting were used as references to predict the odds of getting treatment for AUD based on age, marital status, employment status, education, primary source of income, race, and type and setting of treatment received respectively.

## Results

In Table 1, we report the characteristics of the NJ study population and the proportion of the study population who reported alcohol use based on the independent variables. Of the 9,69,788 participants enrolled in the study, the majority (66.6%) were aged 25-49 years while 19.7% and 13.7% were >50 years, and 12-24 years old, respectively. Males constituted 67.9% of the study population, while 32.1% were females. The racial groups represented in the study sample are Alaska Native (0.4%), American Indian (0.9%), Asian or Pacific Islander (23.7%), Black or African American (74.5%), and White (0.5%). Around 21.4% were full-time employees, 7.9%, 23.4%, and 43.3% were part-time employees, unemployed and not in the labor force, respectively (Table 1). Around 73.7% of the population have never been married, while 11.8% are now married and 14.5% are separated. The participants were further categorized based on reported alcohol use (Table 2). The prevalence of alcohol use is 17.7%, 9.5%, and 2.2% for age groups 25-49, >50, and 12-24 years, respectively. Black or African American has a prevalence of 22.9%, Alaska Native 0.2%, American Indian 0.4%, Asian or Pacific Islander 5.9%, and White 0.1% alcohol use (Table 2).

**Table 1:** Characteristic of the study population (n=969788). Substance Abuse and Mental Health Services Administration, Treatment Episode Data Set (TEDS) Discharges, 2018. Rockville, MD: Substance Abuse and Mental Health Services Administration, 2020.

Variable	n	Percent (%)
Age		
12-24	105531	13.7
25-49	512514	66.6
>50	151743	19.7
Race		
Alaska Native	3158	0.4
American Indian	7269	0.9
Asian or Pacific Islander	180144	23.7
Black or African American	566857	74.5
White	3442	0.5
Gender		
Male	522154	67.9
Female	247388	32.1
Employment		
Full-time	160933	21.4
Part-time	59204	7.9
Unemployed	205455	23.4
Not in labor force	325295	43.3
Marital status		
Never Married	561732	73.7
Now Married	89651	11.8
Separated	110262	14.5
Education		
Less than one school grade, no schooling, nursery school, or kindergarten to Grade 8	33350	4.4
Grades 9-11	162685	21.6
Grade 12 or GED	409208	54.3
1-3 years of college, university or vocational school	103071	13.7
4 years of college, university, BA/BS, some postgraduate study, or more	45731	6
Source of income/support		
Wages/ salary	204910	31.3
Public assistance	63747	9.7
Retirement/pension, disability	44026	6.7
Other	56439	8.6
None	172598	26.3
Type of treatment/setting		
Detox, 24-h, hospital inpatient	55142	12.4
Detox, 24-h, free-standing residential	83931	18.9
Rehab/residential, hospital(non-detox)	304714	68.7

**Table 2:** Study population stratified by alcohol use status.

	Alcohol use status				
	Not reported		Reported		
Variable	n	Percent	n	Percent (%)	Prevalence (%)
Age					
12-24	88333	83.7	17198	16.3	2.2
25-49	375963	73.4	136551	26.6	17.7
>50	78898	52	72845	48	9.5
Race					
Alaska Native	3158	64.5	1122	35.5	0.2
American Indian	7269	62.5	2725	37.5	0.4
Asian or Pacific Islander	180144	74.9	45196	25.1	5.9
Black or African American	566857	69.3	174214	30.7	22.9
White	3442	74.1	892	25.9	0.1
Gender					
Male	522154	69.5	159100	30.5	20.7
Female	247388	72.8	67419	27.3	8.8
Employment					
Full-time	86418	53.7	74515	46.3	9.9
Part-time	39546	66.8	19658	33.2	2.6
Unemployed	154250	75.1	51205	24.9	6.8
Not in labor force	247767	76.2	77528	23.8	10.3
Marital status					
Never married	429505	76.5	132227	23.5	17.4
Now married	45467	50.7	44184	49.3	5.8
Separated	62087	56.3	48175	43.7	6.3
Education					
Less than one school grade, no schooling, nursery school, or kindergarten to Grade 8	23925	71.7	9425	28.3	1.3
Grades 9-11	130830	80.4	31855	19.6	4.2
Grade 12 or GED	288592	70.5	120616	29.5	16
1-3 years of college, university, or vocational school	67059	65.1	36012	34.9	4.8
4 years of college, university, BA/BS, some postgraduate study, or more	20071	43.9	25660	56.1	3.4
Source of income/support					
Wages/salary	119133	58.1	85777	41.9	13.1
Public assistance	47117	73.9	16630	26.09	2.5
Retirement/pension, disability	28337	64.4	15689	35.6	2.4
Other	41211	73	15228	27	2.3
None	136358	79	36240	21	5.5
Type of treatment/setting					
Detox, 24-h, hospital inpatient	39044	70.8	16098	29.2	3.6
Detox, 24-h, free-standing residential	60916	72.6	23015	27.4	5.2
Rehab/residential, hospital (non-detox)	214201	70.3	90513	29.7	20.4

In terms of employment status, the full-time employee, the part-time employees, the unemployed, and those not in the labor force have alcohol use prevalence of 9.9%, 2.6%, 6.8%, and 10.3% respectively (Table 2). The study participants who never married have an alcohol use prevalence of 17.4%, those who are now married have a prevalence of 5.8% while those who are separated have a prevalence of 6.3%. Those with less than one school grade, no schooling, nursery school, or kindergarten to Grade 8 have an alcohol use prevalence of 1.3%, prevalence of 4.8% is seen in those with 1-3 years of college, university, or vocational school, 3.4% in those with 4 years of college, university, BA/BS, some postgraduate study, or more while prevalence of 4.2% and 16% are seen in those who have grades 9-11 and grade 12 or GED, respectively. Considering the source of income or support of the study participants, alcohol use prevalence is 13.1% for those on wages or salary, 2.5% for public assistance, 2.4% for those who have retired, on pension or disability, 2.3% for other source of income while those with no source of income has a prevalence of 5.5%. Of the population who reported alcohol use, the prevalence of receiving detoxification treatment in a 24-h hospital inpatient setting is 3.6%, the prevalence of having detoxification treatment in a 24-h free-standing residential setting is 5.2%, while the prevalence of receiving rehabilitation treatment in a residential, hospital (non-detoxification) setting is 20.4% (Table 2).

The odds of getting treatment for AUD for the age group 15-17 and >50 years when compared to the age group 12-24 years are 1.38, 95% CI [1.33,1.43] and 2.25, 95% CI [2.15, 2.36] respectively (Table 3). The Alaska Native has an OR of 1.67, 95% CI [1.42, 1.96], the American Indian has an OR of 2.12, 95% CI [1.95, 2.31]), Black or African American has an OR of 1.7, 95% CI [1.65-1.75], and the White has an OR of 1.31, 95% CI [1.12-1.52] when compared with the Asian or Pacific Islander (Table 3). When compared to those who completed less than one school grade, no schooling, nursery school, or kindergarten to grade 8, those who completed 4 years of college, university, BA/BS, or some postgraduate study have an odd of getting treatment for AUD of 1.68, 95% CI [1.57, 1.81], those with grades 9-11 have odds of 0.61, 95% CI [0.57, 0.65], grade 12 or GED has odds of 0.85, 95% CI [0.80-0.90], those with 1-3 years of college or vocational school have odds of 1.08, 95% CI [1.57, 1.81] (Table 3).

**Table 3:** Logistic regression predicting treatment in alcohol use disorder based on age, education, marital status, employment status, race, primary source of income, and the type of treatment or setting at admission. ^a^Age comparing each group to 12-24 years; ^b^Education status comparing each group to less than one school grade, no schooling, nursery school, or kindergarten to Grade 8; ​​​​​​​^c^Marital status comparing each group to never married; ​​​​​​​^d^Employment status comparing each group to unemployed; ​​​​​​​^e^Race category comparing each group to Asian or Pacific Islander; ​​​​​​​^f^Primary source of income or support comparing each group to those on salary/wages; ​​​​​​​^g^Type of treatment/service setting at admission comparing each group to rehabilitation/residential, hospital (other than detoxification)

Effect	Point estimate	Confidence limits
Age		
25-49 years^a^	1.38	1.33-1.43
>50 years^a^	2.25	2.15-2.36
Education Status		
Grades 9-11^b^	0.61	0.57-0.65
Grades 12 (or GED)^b^	0.85	0.80-0.90
1-3 years of college, university, or vocational school^b^	1.08	1.01-1.15
4 years of college, university, BA/BS, some postgraduate study or more^b^	1.68	1.57-1.81
Marital status		
Now married^c^	1.61	1.56-1.67
Separated^c^	1.54	1.48-1.59
Employment status		
Full-time^d^	1.62	1.53-1.71
Part-time^d^	1.18	1.11-1.25
Not in labor force^d^	0.89	0.87-0.93
Race		
Alaska Native (Aleut, Eskimo, Indian)^e^	1.67	1.42-1.96
American Indian (other than Alaska Native)^e^	2.12	1.95-2.31
Black or African American^e^	1.7	1.65-1.75
White^e^	1.31	1.12-1.52
Primary source of income/support		
Public assistance^f^	0.91	0.85-0.97
Retirement/pension, disability^f^	0.88	0.82-0.94
Other^f^	0.92	0.87-0.97
None^f^	0.73	0.69-0.76
Type of treatment or service setting at admission		
Detoxification, 24-h service, hospital inpatient^g^	0.88	0.82-0.95
Detoxification, 24-h service, free-standing residential^g^	1.23	1.19-1.29

A full-time employee has an odd of 1.62, 95% CI [1.53, 1.71], the part-time employee has an odd of 1.18, 95% CI [1.11, 1.25], while subjects not in the labor force, including students, retirees, inmates, and disabled (OR= 0.89, 95% CI [0.87-0.93]), when compared to the unemployed. In terms of primary income, and the source of support in getting treatment for AUD, logistic regression shows that those who depend on public assistance have an odd of 0.91, 95% CI [0.85, 0.97], those who are retired, on pension or disability has odd of 0.88, 95% CI [0.82-0.94], those with other sources of income has odd of 0.92, 95% CI [0.87,0.97], and those with no income has odd of 0.73, 95% CI [0.69-0.76] when compared to those on salary or wages (Table 3). The study participants who are married have AUD treatment odd of 1.61, 95% CI [1.56, 1.67] while those who are separated have an odd of 1.54, 95% CI [1.48, 1.59] when compared to those who are never married (Table 3). In terms of the kind of treatment received or service setting at admission for those with AUD in NJ, the odds of receiving detoxification from a 24-h inpatient hospital setting is 0.88, 95% CI [0.82-0.95] while the odds of receiving treatment in a 24-h service free-standing detoxification unit is 1.23, 95% CI [1.19, 1.29] when compared to treatment in a rehabilitation/residential-hospital (other than detoxification) (Table 3).

## Discussion

This study shows that disparity exists for AUD treatment in NJ across age, race, educational status, marital status, source of income or support, and the type of treatment or service setting at admission. There is a significant difference in the odds of getting treatment for AUD between the Asian or Pacific Islander and Alaska Native (1.67, 95% CI [1.42, 1.96]), American Indian (OR= 2.12, 95% CI [1.95, 2.31]), Black or African American (OR= 1.7, 95% CI [1.65-1.75]), and the White (OR =1.31, 95% CI [1.12-1.52]) racial groups. It is striking to note that African- Americans have higher odds of getting treatment for AUD than White when compared with Asian or Pacific Islanders. These findings contrast with a study conducted by Martin et al. (2022) on the receipt of treatment for AUD in the United States, which reported that non-Latinx Blacks had half the odds of receiving treatment than non-Latinx Whites [11]. Additionally, most studies show that African American and Hispanic patients were significantly less likely to complete treatment than their White counterparts [6, 12].

The analysis based on the educational status of the subjects shows that there is a higher odds of getting treatment for AUD in those who completed 4 years of college, university, BA/BS, or some postgraduate study (OR=1.68, 95% CI[1.57,1.81]) compared to those who completed less than one school grade, no schooling, nursery school or kindergarten to grade 8 (Table 3). This finding agrees with the study done by Martin et al. (2022), where the odds of getting treatment are highest in those with education above high school, followed by those with High school/GED education, and the least was those without high school diplomas [11]. Furthermore, a study that analyzed factors affecting outpatient and intensive outpatient alcoholic admissions in NJ indicates that the unskilled have higher odds of dropping out of treatment than the skilled [13]. However, this study highlights that those with grades 9 to 11 (OR=0.61, 95% CI [0.57, 0.65]) and grade 12 or GED (OR=0.85, 95% CI [0.80-0.90]) educational status are less likely to get treatment for AUD compared to those with grade 8 or lesser education. There is also a significant difference in treatment between those with 1-3 years of college or vocational school and those with less than grade 8 or lesser education (see Table 3).

Employment status is another factor reported to impact the receipt of treatment for AUD [14-15]. This study shows that full-time (OR= 1.62, 95% CI [1.53, 1.71]) and part-time (OR=1.18, 95% CI [1.11, 1.25]) employees are more likely to get treatment compared to the unemployed. However, subjects who were not in the labor force, including students, retirees, inmates, and the disabled, were less likely to get treatment for AUD compared to the unemployed. This aligns with the findings by Mennis and Stahler (2016) in their study on ethnic and racial disparity in substance use disorder treatment that stated that the odds of getting treated were higher in those who are full-time or part-time employees than those who are unemployed [15]. Additionally, the treatment completion rate is 7% higher for the employed than for the unemployed [15]. On the contrary, Honkonen et al. (2017) examined the association between employment status, AUDs, and service use for this disorder, and it was reported that the odds for treatment contact were 3.51 times higher for the unemployed than for the employed [14].

Regarding primary income and the source of support in getting treatment for AUD, this study shows that those who depend on public assistance (OR=0.91, 95% CI [0.85, 0.97]), those who are retired, on pension, or disability (OR=0.88, 95% CI [0.82-0.94]), those with other sources of income (OR=0.92, 95% CI [0.87, 0.97]) or those with no income (OR=0.73, 95% CI [0.69-0.76]) were less likely to get treatment when compared to those on salary or wages (Table 3). However, a study by Mennis and Stahler (2016) on the predictors of treatment utilization in treatment naïve adults with AUD highlighted that those with lower income were more likely to receive treatment for AUD [15]. Disparity examined based on marital status shows that subjects who are married (OR=1.61, 95% CI [1.56, 1.67]) and those who are separated (OR=1.54, 95% CI [1.48, 1.59]) are more likely to get treatment when compared to those who are never married. This conflicts with evidence in the literature that reported a higher odd of treatment in unmarried women [16].

In terms of the type of treatment received or service setting at admission for those with AUD in NJ, subjects are less likely to be enrolled in a 24-h service inpatient detoxification unit (OR=0.88, 95% CI [0.82-0.95]) compared to being enrolled in a 24-h service, free-standing residential detoxification unit. However, the odds of receiving treatment in a 24-h service free-standing detoxification unit is higher than treatment in a rehabilitation or residential- hospital (other than detoxification) unit (OR=1.23, 95% CI [1.19, 1.29]). A body of evidence suggests that the treatment modality and setting influence the completion of treatment for AUD. According to Saloner and Lê Cook (2013), the alcohol treatment completion rate is higher for people discharged from residential settings than those discharged from intensive outpatient settings. Those who were discharged from non-intensive outpatient settings have the lowest rates [6].

Limitations and strength

One of the study's limitations is the cross-sectional design nature of our study, as causality cannot be estimated. In addition, some patients who underwent AUD treatment might not have been reported by the facility, which could be responsible for the disparity based on socioeconomic status. A strength of the study is the large sample size, including patients from different socioeconomic backgrounds, thus increasing the external validity and generalizability of the study.

## Conclusions

Our findings highlight inequality in treating AUD in NJ based on the patient’s sociodemographic background, the type of treatment received, and the setting where it was received in NJ. This may have some policy implications involving implementing policies to ensure easy access to treatment and retention in treatment for individuals with AUD, irrespective of their sociodemographic status. In addition, implementing a policy to support a structured referral system to an appropriate treatment setting for the required treatment option will ensure equality in the kind of treatment received for AUD in NJ. Furthermore, providing the necessary social and financial support, including public funding for those who are socially and financially incapable, could drive access to treatment and help reduce or alleviate the observed treatment disparity. Furthermore, a structured follow-up plan, especially for at-risk patients, ensures they follow through on treatment referrals. It is imperative that interventions are targeted toward the at-risk populations and ensure that policies exist to drive access to treatment to all who need it at the local and state levels. Future research should evaluate the effectiveness of any interventions implemented to mitigate the treatment disparity issue. 
